# Supplementary material for: Heterogeneity of synonymous substitution rates in the Xenopus frog genome
Source: PLoS One. 2020 Aug 7;15(8):e0236515. doi: 10.1371/journal.pone.0236515 (PMC7413554; doi:10.1371/journal.pone.0236515)
Supplement: S1 Fig — Representative plots are presented in Fig 2. Centromere locations (vertical dotted line) are based on [15, 16]. (DOCX) [file pone.0236515.s001.docx]

**S1 Fig.** **Relationship between proportion of synonymous substitutions (*k*) and location within each chromosome, for each genome pair combination** (XTR-XLA.L in red, XTR-XLA.S in green, and XLA.L-XLA.S in blue). Representative plots are presented in Fig 2. Centromere locations (vertical dotted line) are based on [15,16].
