# Supplementary material for: Heterogeneity of synonymous substitution rates in the Xenopus frog genome
Source: PLoS One. 2020 Aug 7;15(8):e0236515. doi: 10.1371/journal.pone.0236515 (PMC7413554; doi:10.1371/journal.pone.0236515)
Supplement: S2 Fig — Y-axes represent CG composition in 1Mb sliding windows with a 100kb step on the chromosomes, X-axes represent chromosome locations (x1000000). The dinucleotide composition was computed using a batch-learning self-organizing map (BLSOM) program (Abe et al. 2003). The BLSOM program can be obtained from UNTROD, Inc. (y_wada@nagahama-i-bio.ac.jp). (DOCX) [file pone.0236515.s002.docx]

**S2 Fig. Frequency of CG dinucleotides along each chromosome based on whole genome data of *Xenopus tropicalis* and *X. laevis* L and** **S**. Y-axes represent CG composition in 1Mb sliding windows with a 100kb step on the chromosomes, X-axes represent chromosome locations (x1000000). The dinucleotide composition was computed using a batch-learning self-organizing map (BLSOM) program (Abe et al. 2003). The BLSOM program can be obtained from UNTROD, Inc. (y_wada@nagahama-i-bio.ac.jp).

**
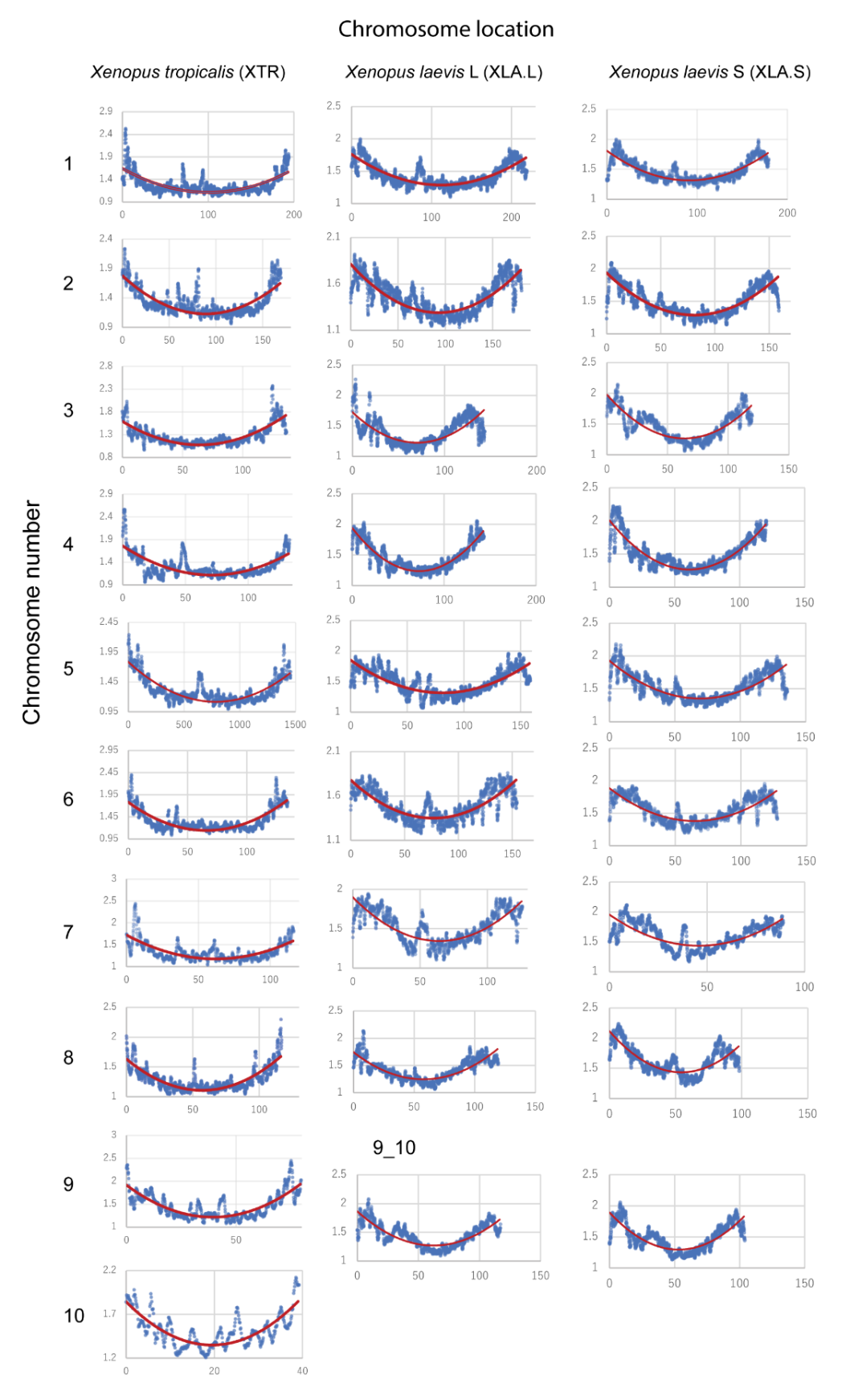
**
