# Supplementary material for: Heterogeneity of synonymous substitution rates in the Xenopus frog genome
Source: PLoS One. 2020 Aug 7;15(8):e0236515. doi: 10.1371/journal.pone.0236515 (PMC7413554; doi:10.1371/journal.pone.0236515)

**S3 Fig. Unique patterns observed between proportion of synonymous substitutions *k* and location** within (A) chromosome 8, (B) chromosome 3 (normal pattern), and (C) chromosome 9/10 could be attributed to chromosomal rearrangements previously identified using cytogenetic mapping by Session et al. [5].


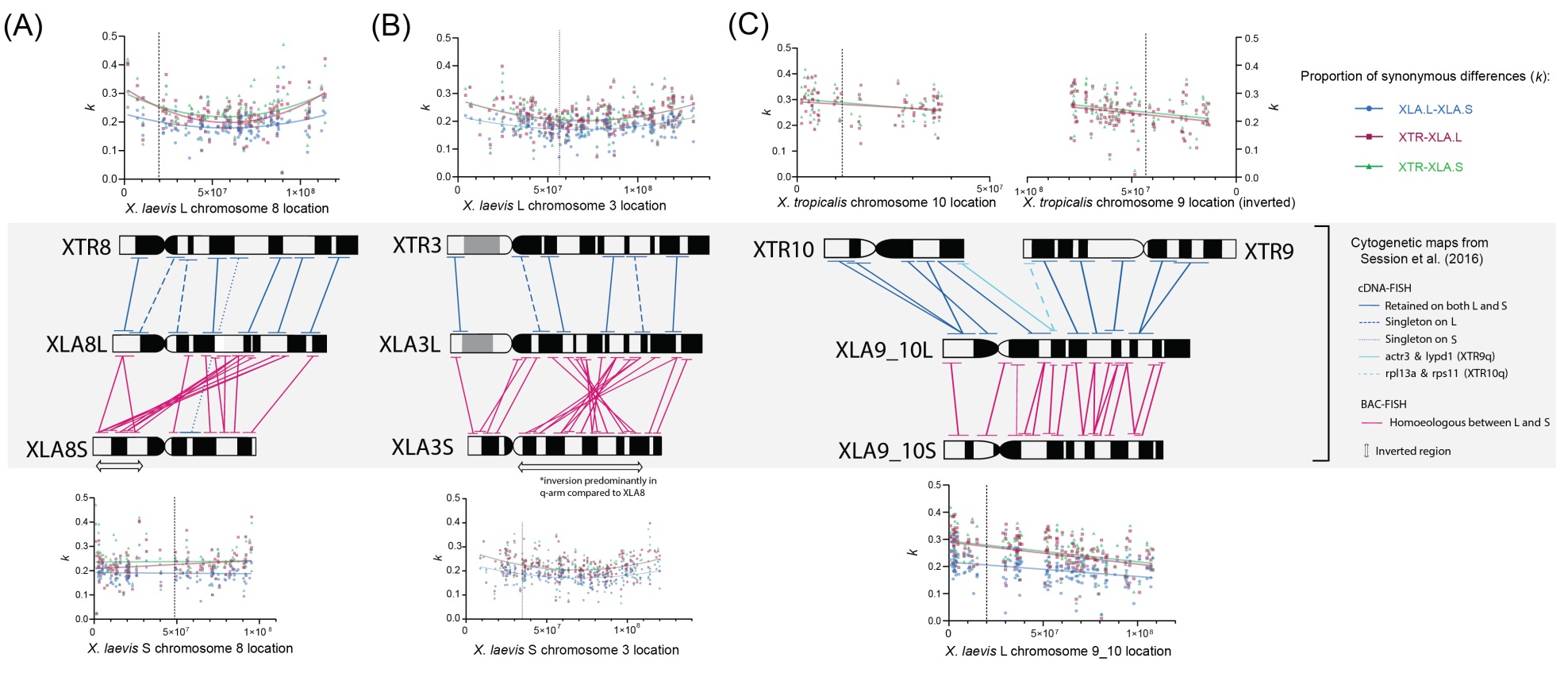

Supplement: S3 Fig — Unique patterns observed between proportion of synonymous substitutions k and location within (A) chromosome 8, (B) chromosome 3 (normal pattern), and (C) chromosome 9/10 could be attributed to chromosomal rearrangements previously identified using cytogenetic mapping by Session et al. [5]. (DOCX) [file pone.0236515.s003.docx]
