# Supplementary material for: Heterogeneity of synonymous substitution rates in the Xenopus frog genome
Source: PLoS One. 2020 Aug 7;15(8):e0236515. doi: 10.1371/journal.pone.0236515 (PMC7413554; doi:10.1371/journal.pone.0236515)
Supplement: S1 Table — Partial F-test was used to compare two models: linear [lm(W~Z)] and quadratic [lm(W~Z + I (Z^2)]. Values presented are significance probabilities associated with the F values, Pr(F), and were calculated independently for each pairwise comparison of genomes: XLA.L-XLA.S (blue), XTR-XLA.L (red), and XTR-XLA.S (green). Values with Pr(F) < 0.01 (in bold) significantly support a quadratic model. (DOCX) [file pone.0236515.s004.docx]

**S1 Table.** **Quadratic regression analyses of relationship between *W* = chromosome location and *Z* = proportion of synonymous substitutions (*k*).** Partial *F*-test was used to compare two models: linear [lm(*W~Z*)] and quadratic [lm(*W~Z* + I (*Z*^2)]. Values presented are significance probabilities associated with the *F* values, *Pr(F)*, and were calculated independently for each pairwise comparison of genomes: XLA.L-XLA.S (blue), XTR-XLA.L (red), and XTR-XLA.S (green). Values with *Pr(F)* < 0.01 (in bold) significantly support a quadratic model.

| Chromosome # | *Pr(F)*^a^ | *Pr(F)*^b^ | *Pr(F)*^c^ |
| --- | --- | --- | --- |
| 1 | **6.667e-05**  **2.88e-13**  **7.601e-13** | **1.955e-05**  **2.668e-13**  **2.523e-13** | **2.081e-05**  **4.549e-13**  **2.882e-13** |
| 2 | **0.002032**  **6.846e-10**  **3.753e-08** | **0.002574**  **5.204e-10**  **2.498e-08** | **0.003172**  **1.068e-05**  **4.693e-05** |
| 3 | **0.001713**  **0.0002333**  **0.0009391** | **5.284e-05**  **9.002e-07**  **9.572e-06** | **0.0002013**  **5.705e-06**  **9.185e-05** |
| 4 | **1.505e-05**  **3.839e-10**  **8.814e-08** | **1.034e-08**  **5.468e-14**  **1.113e-11** | **5.362e-08**  **5.362e-08**  **6.203e-09** |
| 5 | **0.000486**  **1.67e-08**  **1.089e-07** | **6.812e-05**  **1.069e-08**  **2.87e-07** | **3.167e-05**  **9.205e-05**  **2.449e-06** |
| 6 | **0.008141**  **9.479e-08**  **2.722e-09** | **0.007303**  **1.293e-07**  **3.097e-09** | **0.01495 n.s.**  **1.017e-06**  **1.604e-08** |
| 7 | 0.1015 n.s.  **1.685e-06**  **1.778e-08** | 0.155 n.s.  **2.262e-06**  **1.793e-07** | 0.1619 n.s.  **2.274e-06**  **1.143e-07** |
| 8 | **0.003185**  **2.935e-09**  **4.949e-05** | **0.002904**  **2.01e-09**  **4.847e-05** | 0.2093 n.s.  0.1641 n.s.  0.08051 n.s. |
| 9 | 0.06295 n.s.  0.04673 n.s. | - | - |
| 10 | 0.1918 n.s.  0.03979 n.s. | - | - |
| 9_10 | - | 0.052 n.s.  0.9725 n.s.  0.6253 n.s. | 0.3499 n.s.  0.6411 n.s.  0.7788 n.s. |

Chromosome location based on ^a^XTR, ^b^XLA.L, or ^c^XLA.S
