# Supplementary material for: Heterogeneity of synonymous substitution rates in the Xenopus frog genome
Source: PLoS One. 2020 Aug 7;15(8):e0236515. doi: 10.1371/journal.pone.0236515 (PMC7413554; doi:10.1371/journal.pone.0236515)
Supplement: S2 Table — Although chromosome 9_10 is homoeologous in XLA.L and XLA.S, we separated estimations to chromosome 9 or 10 based on location in XTR. (DOCX) [file pone.0236515.s005.docx]

**S2 Table.** **Maximum likelihood estimates and calculations of rate heterogeneity (α), ancestral population size (N_A_), and divergence time (t).** Although chromosome 9_10 is homoeologous in XLA.L and XLA.S, we separated estimations to chromosome 9 or 10 based on location in XTR.

| Chrom-osome | α (estimated with X = 0) | | |  | α (with best ML estimates of X and Y) | | |  | N_A_ (x10^6^)* | | |  | T (mya)* | | |
| --- | --- | --- | --- | --- | --- | --- | --- | --- | --- | --- | --- | --- | --- | --- | --- |
|  | XLA.S-XLA.L | XTR-XLA.L | XTR-XLA.S |  | XLA.S-XLA.L | XTR-XLA.L | XTR-XLA.S |  | XLA.S-XLAL | XTR-XLA.L | XTR-XLA.S |  | XLA.S-XLAL | XTR-XLA.L | XTR-XLA.S |
| 1  2  3  4  5  6  7  8  9  10 | 20.8  31.0  41.8  28.0  23.8  23.0  28.9  22.6  18.5  21.4 | 22.3  30.0  34.3  20.3  19.2  19.2  27.4  17.6  15.2  39.3 | 22.4  33.4  29.9  20.8  24.1  19.1  31.0  18.6  14.9  37.8 |  | 15  25  35  35  24  23  29  22  18  21 | 22  30  40  20  19  15  28  18  16  30 | 22  40  30  21  24  15  31  19  11  38 |  | 0.88  1.11  1.08  2.58  1.78  1.13  1.86  2.52  1.55  1.27 | 2.61  1.66  2.82  2.86  2.69  2.12  2.56  3.66  0.96  1.35 | 2.82  2.85  2.31  2.78  2.19  1.98  1.36  4.02  2.62  1.92 |  | 41.2  42.9  41.6  38.7  40.7  40.4  41.4  40.9  47.0  39.0 | 45.5  51.1  47.0  48.0  52.4  50.9  51.9  44.8  64.3  51.4 | 49.2  50.6  48.7  50.6  54.3  52.1  55.3  48.9  62.6  52.1 |

*N_A_ and T were calculated assuming µ = 2.05 x 10^-9^
