# Supplementary material for: Heterogeneity of synonymous substitution rates in the Xenopus frog genome
Source: PLoS One. 2020 Aug 7;15(8):e0236515. doi: 10.1371/journal.pone.0236515 (PMC7413554; doi:10.1371/journal.pone.0236515)
Supplement: S3 Table — (DOCX) [file pone.0236515.s006.docx]

**S3 Table.** **Chi-square p-values following fitting observed data (synonymous difference) against the distribution 'nbinom' by maximum likelihood.**

| Chromosome | XLA.L-XLA.S | XLA.L-XTR | XLA.S-XTR |
| --- | --- | --- | --- |
| 1 | 0.096 | 0.044 | 0.020 |
| 2 | 0.115 | 0.874 | 0.355 |
| 3 | 0.067 | 0.353 | 0.319 |
| 4 | 0.337 | 0.548 | 0.137 |
| 5 | 0.078 | 0.229 | 0.038 |
| 6 | 0.094 | 0.544 | 0.338 |
| 7 | 0.444 | 0.143 | 0.338 |
| 8 | 0.344 | 0.051 | 0.443 |
| 9 | 0.481 | 0.467 | 0.499 |
| 10 | 0.403 | 0.719 | 0.187 |
| All | **3.26E-08** | **2.06E-06** | **6.38E-09** |
